# Development, Translation, and Validation of the Japanese Quality of Dying and Death Questionnaire for Families of ICU Patients

**DOI:** 10.7759/cureus.77161

**Published:** 2025-01-08

**Authors:** Kazuaki Naya, Hideaki Sakuramoto, Yuki Kuroiwa, Rika Hamano, Chihiro Kawaguchi, Hina Yamamoto, Wakana Sugihara, Kyoka Horita, Ami Nakaue, Hiromi Iwashita

**Affiliations:** 1 Department of Adult Nursing, Wakayama Faculty of Nursing, Tokyo Health University, Wakayama, JPN; 2 Department of Critical Care and Disaster Nursing, Japanese Red Cross Kyushu International College of Nursing, Munakata, JPN; 3 Department of Intensive Care Medicine, Japanese Red Cross Wakayama Medical Center, Wakayama, JPN

**Keywords:** end-of-life care, intensive care unit, palliative care, quality of dying and death questionnaire, validation study

## Abstract

Background and aim: Intensive care unit (ICU) mortality rates are notably high. Several studies outside Japan indicate that the Quality of Death and Dying (QODD) in ICUs is often rated lower compared with settings such as hospices or specialized palliative care units. However, there are no tools to directly evaluate QODD in ICUs that are appropriate to Japanese culture and medicine. Consequently, no study has been conducted in Japanese ICU settings. This study aimed to develop a Japanese version of the QODD Questionnaire 3.2A (ICU-QODD) and to evaluate its reliability and validity.

Methods: The ICU-QODD was translated into Japanese following the guidelines for translation, adaptation, and validation of instruments. A pilot test involving an expert panel and family members of 40 ICU survivors helped establish the content validity index (CVI). Further testing with 59 bereaved family members evaluated validity through convergent and known groups analyses, while reliability was assessed through internal consistency and test-retest methods.

Results: The pilot test confirmed the face and content validity of the Japanese ICU-QODD. Following revisions, all items achieved a CVI of 0.78 or higher, with an average scale-level CVI exceeding 0.90. One item with low CVI was excluded, resulting in a finalized 24-item version. The ICU-QODD demonstrated strong construct validity, with significant domain correlations to the total score. Known-groups analysis revealed lower QODD scores for patients admitted from the ward *(*p=0.03). Internal consistency and reliability were robust, with the intraclass correlation coefficient generally above 0.7 (range: 0.705-0.964, p<0.001).

Conclusions: The Japanese version of the ICU-QODD translated in this study demonstrates satisfactory validity and reliability, making it suitable for clinical use in Japan, and adapted to the country’s medical, cultural, and linguistic context.

## Introduction

The intensive care unit (ICU) is a facility where critically ill patients with life-threatening conditions receive advanced medical technology under 24-h close observation, with the primary purpose of saving their lives. However, owing to the severity of these cases, mortality rates are elevated. Although international comparative data on ICU mortality are limited, the overall mortality rate for adult ICU patients ranges from 10% to 29% [1].

The emphasis on life-saving therapies in intensive care often complicates decisions regarding the withdrawal of care. Advances in life-support technologies, such as extracorporeal membrane oxygenation or ventricular assist devices, have further blurred the boundaries of end-of-life care. Consequently, many healthcare professionals involved in intensive care experience difficulties in providing end-of-life care in the ICU setting [2,3]. Furthermore, several studies have reported that the Quality of Dying and Death (QODD) in ICUs is often lower compared with settings such as hospices or specialized palliative care [4,5].

Therefore, improving QODD in ICUs is a critical concern. Consequently, various interventions have been implemented globally, including palliative care education for ICU clinicians, nurses, and other healthcare providers, as well as the involvement of palliative care specialists in ICU rounds [6].

However, research focusing on improving ICU patients’ QODD in Japan remains limited. This gap is largely owing to the absence of validated tools to measure QODD in Japanese ICU settings. The QODD Questionnaire 3.2A (ICU-QODD) is the most widely used tool globally for assessing QODD in the ICU setting [7,8]. The ICU-QODD has been well-evaluated for validity and reliability [9]. Its availability in Japan would be the first step toward improving the quality of end-of-life care by enabling the assessment of QODDs in ICU patients. Therefore, it is necessary to develop an ICU-QODD adapted to Japanese culture and healthcare systems. To this end, this study aimed to translate the ICU-QODD into the Japanese language and evaluate its validity and reliability [10].

## Materials and methods

Study design

This methodological study involved translating the ICU-QODD into the Japanese language (translation process) and evaluating its validity and reliability with bereaved families (validity and reliability process) according to established guidelines [11].

Translation process

Permission to translate the original questionnaire (ICU-QODD) was obtained from Elizabeth L. Nielsen, one of its developers. The ICU-QODD consists of 25 items (appendix 1) [12]. The questionnaire includes 22 items assessing aspects of the respondents’ experience during their loved one’s final days, two items evaluating the quality of care provided by the medical team, and one item assessing the overall quality of the death experience. The 22 items are divided into six domains as follows: (1) symptoms and personal care, (2) preparation for death, (3) moment of death, (4) family, (5) treatment preferences, and (6) whole person concerns. Each of these 22 items consists of two parts as follows: (a) an evaluation of frequency or presence (0 = none to 5 = always or yes, no) and (b) a rating of the patient’s dying experience (0 = terrible to 10 = almost perfect). The total score is calculated by summing the ratings for each item, dividing this by the number of answered items, then dividing by 10 and multiplying by 100; higher scores indicate a better QODD.

**Figure 1:**
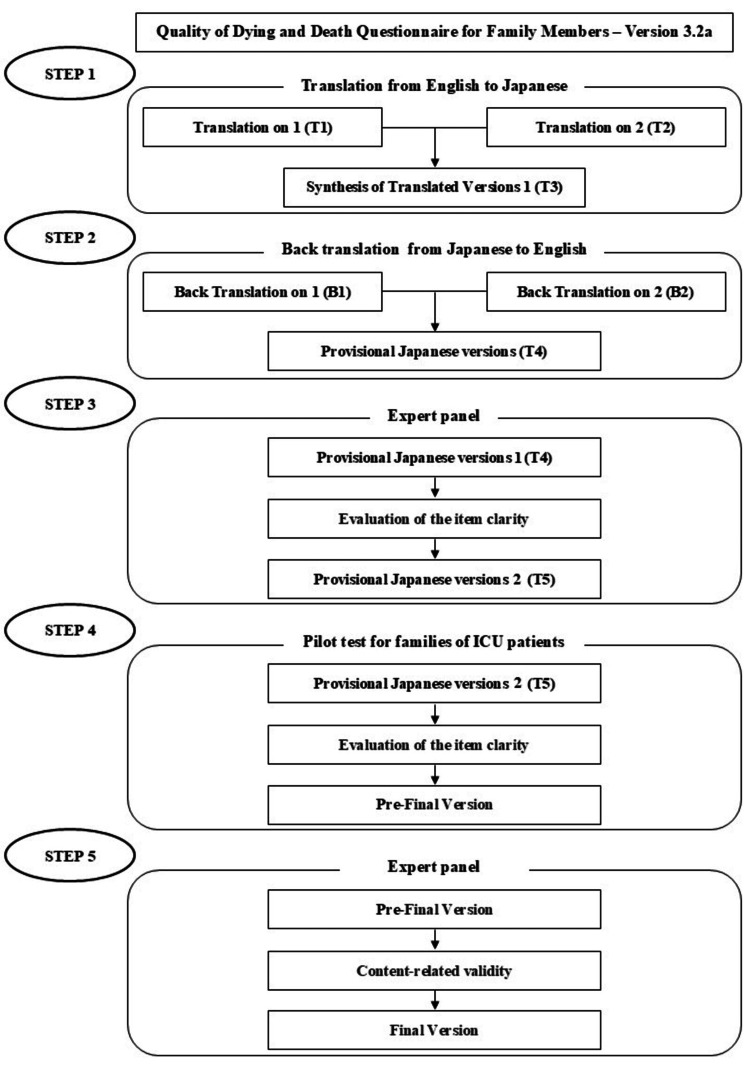
Translation and cultural adaptation process into Japanese.

Following the back-translation method and previously established guidelines, a translation team consisting of two nursing lecturers, four clinical nurses, and one of the original authors participated in the process [11]. The process included five steps, including face validation and content validation, and was conducted from May 2023 to February 2024 (Figure 1).

Step One

Two nursing lecturers independently translated the original ICU-QODD from English to Japanese language, producing translations T1 and T2, which were subsequently combined into version T3 after discussions between the translators. Equivalence between the original and translated versions was assessed by two nursing lecturers, who evaluated clarity for healthcare professionals and the patient's family.

Step Two

Two translators, blinded to the original scale, independently back-translated versions T3 from Japanese to English language, resulting in versions B1 and B2. One translator was a native English speaker with medical knowledge, while the other was a native English speaker with no medical background. Two nursing lecturers, along with one of the original authors, evaluated equivalence between the original, B1, and B2, adjusting to minimize linguistic discrepancies. After refinements, a provisional Japanese version, T4, was developed.

Step Three

An expert panel consisting of three clinical nurses, three nursing college faculty, one medical professional who had experienced family loss in the ICU, two clinical psychologists, one ICU physician, and one palliative care physician evaluated each item in T4 for “clarity” or “unclearness” through an online survey. Experts with diverse backgrounds were selected to modify the content and representation to be appropriate for the Japanese culture and healthcare system. The expert panel should consist of six to 10 members who are knowledgeable about the content areas of the measure’s components and the target population for which the measure is intended, and who are native speakers of the measure’s language. The proportion of “unclear” responses was calculated, and participants rated “unclear” items or offered suggestions for improvement.

Step Four

A pilot test of T5 was conducted to assess item clarity. Item clarity is pilot-tested among participants whose language is the target language of the instrument to evaluate the instructions, response format, and the items of the instrument for clarity. A sample size of 10-40 individuals is recommended in the guidelines. Only family members of ICU patients whose conditions had improved and who were being transferred to the ward were included in the study. The pilot test focused on face validity and involved family members of ICU survivors who were less burdened. The test was explained to them before they left the ICU, and their responses were collected by completing and mailing the questionnaire. Consequently, a total of 40 family members of ICU patients evaluated the clarity of the scale using the same process outlined in step three.

Step Five

An expert panel rated each item of the pre-final version on a four-point Likert scale as follows: 1 = not relevant, 2 = unable to assess relevance, 3 = relevant but needs minor revision, 4 = very relevant and concise. The content validity index (CVI) was calculated as the proportion of items rated 3 or 4. A CVI of ≥0.80 was deemed acceptable, with item-level and scale-level content validity indices computed for each item and the entire scale. Revisions continued until item-level and scale-level CVIs reached ≥0.78 and ≥0.90, respectively, culminating in the final Japanese version [11].

Validity and reliability process from the bereaved family

Data were collected from the bereaved family through a postal survey between April and July 2024 to evaluate the validity of the ICU-QODD.

Inclusion criteria were as follows: (1) family members of patients admitted to the ICU as an emergency and who stayed for at least 48 h within six months prior to the survey to the past five years, (2) family members of patients who passed away in the ICU or the ward following ICU discharge within the past five years, and (3) individuals aged 18 years or older. Exclusion criteria included: (1) inability to provide consent, (2) family members who are unaware of the patient’s condition because they never visited the patient in the ICU, (3) individuals who required emotional support, such as counseling, as indicated in medical records, (4) history of mental illness, and (5) non-native Japanese speakers.

Construct validity was assessed by convergent validity through correlation between ICU-QODD scores and QODD-1 scores and by known group validity from previous studies. Initial contact was made by telephone, providing a brief study description and seeking permission to mail study materials. Subsequently, the final version ICU-QODD Questionnaire was mailed, and patient and family baseline characteristics were collected from electronic medical records. The ICU-QODD’s intrarater reliability was evaluated using the test-retest method. Participants agreeing to the test-retest method received the ICU-QODD a second time, two weeks after their initial response, with a reminder sent two weeks after mailing.

Sample size for the validity and reliability process

The sample size for translation was based on established guidelines [11]. For validity and reliability assessment, a minimum of 50 bereaved families were included. Intrarater reliability was determined utilizing the intraclass correlation coefficient (ICC) based on test-retest responses. Following Zou’s formula, a sample size of 41 was calculated assuming a significance level of 0.05 and a power of 0.8 [13]. For construct validity evaluation, correlation coefficients between ICU-QODD scores and a single-item QODD-1 rating were calculated. With an effect size of 0.4, a significance level of 0.05, and a power of 0.8, the required sample size was calculated at 46. Internal consistency testing targeted a sample size of 50 or more, based on prior research [14]. The overall target sample size was therefore set at 50 or more.

Data analysis for the validity and reliability process

Participant characteristics were summarized using descriptive statistics. The normality of each dataset was assessed. Data were expressed as mean±standard deviation (SD), and parametric tests were applied based on the results. Construct validity, internal consistency, and intrarater reliability were assessed to confirm reliability and validity. Convergent validity was evaluated by calculating the correlation coefficient between the ICU-QODD and QODD-1 scores. Internal consistency was measured using Cronbach’s alpha, with values >0.7 indicating satisfactory consistency [15]. Intrarater reliability was evaluated using a two-way random effects ICC model, with ICC <0.5 indicating poor reliability, 0.5-0.75 indicating moderate reliability, 0.75-0.9 indicating good reliability, and ≥0.9 indicating excellent reliability [16]. The t-test was used to compare the groups in the known groups analysis. The ICU-QODD was expected to distinguish between the following groups based on the hypotheses and the following factors: male patients scoring higher than female patients, patients admitted from the emergency room scoring higher than those admitted from the ward, patients who were accompanied by family members at the time of death scoring higher, patients who did not undergo chest compressions at the end of life scoring higher, and patients with advance directives scoring higher [8]. Analyses were performed with IBM SPSS version 28.0 for Windows (Armonk, NY: IBM Corp.).

Ethical considerations

The study protocol was approved by the Ethics Committee of the Japanese Red Cross Wakayama Medical Center (#1144 and #1176). The survey was anonymous, and participants reviewed the consent form and indicated their willingness to participate before completing the questionnaire.

## Results

Translation process

Following approval from the original authors to review the back-translated ICU-QODD, 11 experts evaluated the clarity of the provisional Japanese version (T4). Twenty percent of respondents found the text explanatory and seven items unclear, specifically (4a) How often did your loved one appear to breathe comfortably?, (5a) How often did your loved one appear to feel at peace with dying?, (8a) How often did your loved one appear to keep his/her dignity and self-respect?, (11a) Was your loved one touched or hugged by his/her loved ones?, (12a) Were all of your loved one's healthcare costs taken care of?, (16a) Did your loved one have a spiritual service or ceremony before his/her death?, and (23) Overall, how would you rate the quality of your loved one’s dying? These unclear items were revised over three survey rounds, resulting in Provisional Japanese Version 2 (T5). A pilot test was conducted with family members of ICU survivors, collecting 10 responses to assess comprehensibility. Six items (14a, 15a, 16a, 18a, 19a, and 23) were again rated as “unclear” by more than 20% of respondents. These items were subsequently revised. For example, religious or spiritual ceremonies were adapted into “farewell parties” with less religious overtones. The survey was repeated four times, culminating in a pre-final version based on feedback from 40 participants.

A content validity assessment was then conducted by experts on the pre-final version. Revisions were made until each item’s validity index reached 0.78 or higher, with the average scale-level CVI surpassing 0.90. However, item 12a (“Were all of your loved one’s healthcare costs taken care of?”) received a validity score of 0.10, and this item was excluded from the questionnaire because it was deemed unsuitable for Japanese culture owing to universal health insurance. The finalized Japanese version of the ICU-QODD is presented in appendix 2.

Validity and reliability process

**Figure 2:**
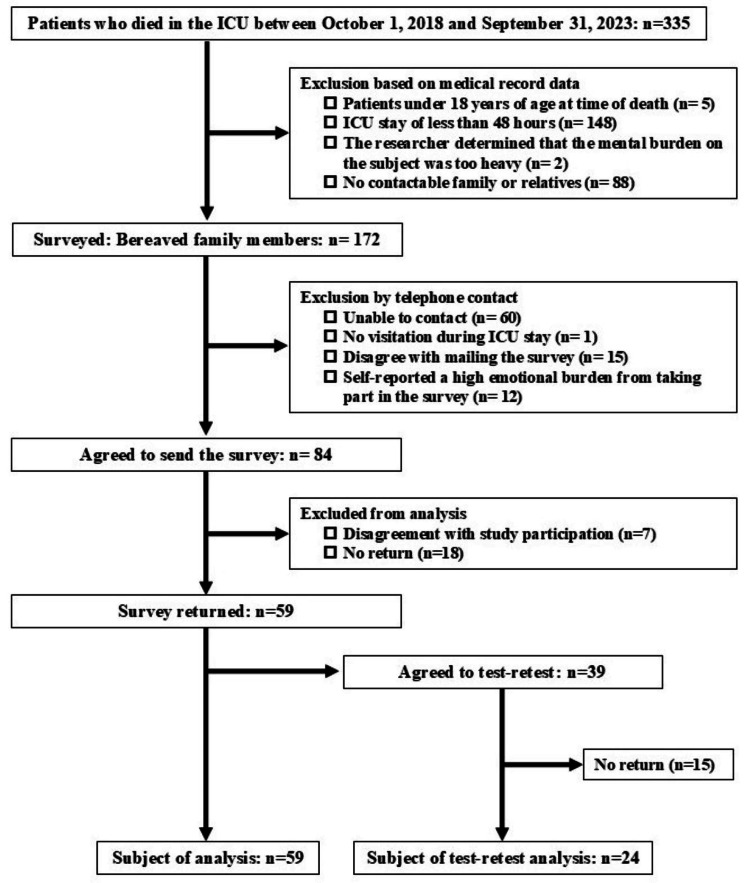
Participants and dropouts in reliability and validity surveys. ICU: intensive care unit

Between October 2018 and September 2023, 335 patients passed away in the ICU. Of these, families of 172 patients meeting the eligibility criteria were contacted by phone, and questionnaires were mailed to 84 bereaved family members who consented to participate. Responses were received from 59 individuals (response rate: 70.2%), with 24 providing responses for the retest (response rate: 61.5%) (Figure 2).

The baseline characteristics of the patients and respondents are presented in Tables 1, 2. Approximately 80% of the respondents’ loved ones were admitted through the emergency department (ED), compared with non-respondents. Only one respondent, among both groups, reported an advance directive. About 70% of respondents were present at the time of their loved one's death.

**Table 1:** Baseline characteristics of patients who died in the ICU. SD: standard deviation; ICU: intensive care unit; ED: emergency department

Variable		Respondents (n=59)	Non-respondents (n=18)
Sex	Male, n (%)	35 (59.3)	9 (50.0)
Age at death (years)	Mean (SD)	74.1 (12.3)	70.9 (14.7)
Clinical department	Intensive care, n (%)	3 (5.1)	2 (11.0)
	Cardiology, n (%)	26 (44.1)	9 (50.0)
	Cardiovascular surgery, n (%)	6 (10.2)	1 (5.6)
	Pulmonology, n (%)	6 (10.2)	0 (0)
	Gastroenterology, n (%)	5 (8.5)	1 (5.6)
	Neurosurgery, n (%)	5 (8.5)	1 (5.6)
	Orthopedics, n (%)	2 (3.3)	1 (5.6)
	Nephrology, n (%)	1 (1.7)	2 (11.0)
	Hematology, n (%)	2 (3.3)	0 (0)
	Dermatology, n (%)	1 (1.7)	1 (5.6)
	Breast surgery, n (%)	1 (1.7)	0 (0)
	Obstetrics and gynecology, n (%)	1 (1.7)	0 (0)
Hospital admission route	ED, n (%)	46 (78.0)	14 (77.8)
	Ward, n (%)	13 (22.0)	4 (22.2)
Ventilator	Yes, n (%)	47 (79.7)	14 (77.8)
Dialysis	Yes, n (%)	11 (19.0)	7 (38.9)
Advance directive	Yes, n (%)	1 (1.7)	0 (0)
Chest compression	Yes, n (%)	4 (6.8)	2 (11.0)
ICU stay (days)	Mean (SD)	8.73 (6.46)	7.67 (3.94)

**Table 2:** Baseline characteristics of bereaved families. SD: standard deviation

Variable		1st test (n=59)	Retest (n=24)
Sex (male)	n (%)	24 (40.7)	14 (58.3)
Age at the time of survey (years)	Mean (SD)	59.6 (12.5)	61.5 (12.9)
Relationship to patient	Spouse/domestic partner, n (%)	20 (33.9)	7 (29.1)
	Parent, n (%)	1 (1.7)	1 (4.2)
	Child, n (%)	33 (55.9)	12 (50.0)
	Sibling, n (%)	2 (3.4)	1 (4.2)
	Other relative, n (%)	3 (5.1)	3 (12.5)
Lived with patient	Yes, n (%)	33 (55.9)	33 (55.9)
Presence at bereavement	Yes, n (%)	41 (69.5)	16 (66.7)
	None, n (%)	17 (28.8)	8 (33.3)
	Unknown, n (%)	1 (1.7)	0 (0)

The construct validity results are presented in Table 3. Positive correlations were observed between each domain score and QODD-1 with the ICU-QODD total score. A known-groups analysis, conducted based on patient and respondent characteristics, indicated significantly lower QODD scores for patients admitted from the ward (p=0.03), reflecting differences between ICU admission routes (ED vs. Ward). No significant differences were found in other groups (Table 4).

**Table 3:** Assessment of construct validity by correlation with total ICU-QODD score. Construct validity was assessed by convergent validity through Pearson correlation between ICU-QODD scores and QODD-1 scores. ICU-QODD: Intensive Care Unit-Quality of Death and Dying (Questionnaire 3.2A); QODD-1: Quality of Death and Dying (a single item rating of the overall quality of dying and death)

Variable	Correlation coefficient	95% CI	p-Value
Symptoms and personal care	0.810	0.689-0.888	<0.001
Preparation for death	0.930	0.878-0.960	<0.001
Moment of death	0.616	0.406-0.765	<0.001
Family	0.797	0.665-0.881	<0.001
Treatment preferences	0.815	0.689-0.893	<0.001
Whole person concerns	0.743	0.580-0.848	<0.001
QODD-1	0.750	0.603-0.848	<0.001

**Table 4:** Known groups comparison of ICU-QODD scores. ICU-QODD: Intensive Care Unit-Quality of Death and Dying (Questionnaire 3.2A); SD: standard deviation; CI: confidence interval; ED: emergency department

Variables	Category	Sample size	QODD score (mean±SD)	95% CI	p-Value
Patient sex	Male	34	52.22 (22.54)	-19.82 to 6.38	0.31
	Female	24	45.50 (27.13)		
Mechanical ventilator	Yes	46	47.73 (24.28)	-7.70 to 24.16	0.31
	None	21	55.97 (25.51)		
Hospital admission route	ED	45	53.27 (23.82)	-32.05 to -2.18	0.03
	Ward	13	36.16 (23.15)		
Chest compressions	Yes	6	50.89 (27.09)	-23.00 to 19.77	0.88
	None	52	49.27 (24.52)		
Advance directive	Yes	1	65.29	-65.99 to 33.72	0.52
	None	57	49.16 (24.67)		
Present at the moment of death	Yes	36	53.72 (23.80)	-25.34 to 3.33	0.13
	None	17	42.72 (25.23)		
Lived with patient	Yes	32	45.18 (22.69)	-3.35 to 22.35	0.14
	None	26	54.68 (26.14)		

Cronbach’s alpha values for internal consistency were calculated, with all domains achieving values above 0.7, indicating good internal consistency (Table 5). The ICU-QODD’s intrarater reliability was evaluated using the test-retest method. Two weeks after the initial responses, the survey was administered again to 24 participants. Item 17, "Did your loved one receive dialysis for his/her kidneys?" demonstrated an ICC below 0.5, while item 18, "Did your loved one have his/her funeral arrangements in order prior to death?" showed an ICC of 0.5252. All other items exhibited ICCs greater than 0.7 (range: 0.705-0.964, p<0.001), indicating good reliability (Table 6).

**Table 5:** Internal consistency of the ICU-QODD. ICU-QODD: Intensive Care Unit-Quality of Death and Dying (Questionnaire 3.2A)

Variable	Cronbach's alpha
ICU-QODD total score	0.995
Symptoms and personal care	0.958
Preparation for death	0.931
Moment of death	0.752
Family	0.880
Treatment preferences	0.920
Whole person concerns	0.760

**Table 6:** Intraclass correlation coefficients for the test-retest method in ICU-QODD items. ICU-QODD: Intensive Care Unit-Quality of Dying and Death (Questionnaire 3.2A); ICC: intraclass correlation coefficient; CI: confidence interval

ICU-QODD item	ICC	95% CI	p-Value
1. How often did your loved one appear to have his/her pain under control?	0.953	0.871-0.983	<0.001
2. How often did your loved one appear to have control over what was going on around him/her?	0.818	0.604-0.922	<0.001
3. How often was your loved one able to feed her/himself?	0.844	0.648-0.935	<0.001
4. How often did your loved one appear to breathe comfortably?	0.902	0.770-0.960	<0.001
5. How often did your loved one appear to feel at peace with dying?	0.964	0.905-0.986	<0.001
6. How often did your loved one appear to be unafraid of dying?	0.940	0.793-0.983	<0.001
7. How often did your loved one laugh and smile?	0.899	0.777-0.956	<0.001
8. How often did your loved one appear to keep his/her dignity and self-respect?	0.705	0.353-0.882	<0.001
9. How often did your loved one spend time with his/her family or friends?	0.813	0.608-0.916	<0.001
10. How often did your loved one spend time alone?	0.828	0.548-0.942	<0.001
11. Was your loved one touched or hugged by his/her loved ones?	0.735	0.453-0.883	<0.001
12. Did your loved one say goodbye to loved ones?	0.913	0.793-0.965	<0.001
13. Did your loved one clear up any bad feelings with others?	0.927	0.753-0.980	<0.001
14. Did your loved one have one or more visits from a religious or spiritual advisor?	0.815	0.533-0.934	<0.001
15. Did your loved one have a spiritual service or ceremony before his/her death?	0.943	0.857-0.977	<0.001
16. Did your loved one receive a mechanical ventilator (respirator) to breathe for him/her?	0.750	0.447-0.898	<0.001
17. Did your loved one receive dialysis for his/her kidneys?	0.494	-0.05-0.796	=0.026
18. Did your loved one have his or her funeral arrangements in order prior to death?	0.525	0.106-0.786	=0.009
19. Did your loved one discuss his or her wishes for end-of-life care with his/her doctor (e.g., resuscitation or intensive care)?	0.773	0.430-0.921	<0.001
20. Was anyone present at the moment of your loved one’s death?	0.858	0.689-0.939	<0.001
21. In the moment before your loved one’s death what was your loved one's condition?	0.814	0.569-0.926	<0.001
22. Overall, how would you rate the quality of your loved one’s dying?	0.845	0.674-0.930	<0.001
23. Rate the care your loved one received from all doctors and other healthcare providers during the last several days of his or her life while in the ICU.	0.848	0.675-0.933	<0.001
24. Rate the care your loved one received from his or her doctor during the last several days of his or her life while in the ICU.	0.754	0.503-0.888	<0.001
ICU-QODD total score	0.958	0.905-0.981	<0.001

## Discussion

This study successfully developed a Japanese version of the ICU-QODD in accordance with established translation guidelines [11]. Both experts and end users evaluated the clarity of the translation, and specialists assessed its content validity, ensuring alignment with Japan’s medical, cultural, and linguistic context. The validity and reliability of the Japanese ICU-QODD were subsequently confirmed through a survey of bereaved family members.

Adaptations were made to the ICU-QODD to suit Japanese medical and cultural practices. Based on expert evaluations of comprehensibility and content validity, and an end-user assessment, revisions were made to certain items, and item (12a) "Were all of your loved one’s healthcare costs taken care of?” was removed. In Japan, the National Health Insurance System ensures that medical costs remain affordable for most of the population. Moreover, a domestic survey on the components of a desirable death did not identify medical costs as a significant factor [17]. Consequently, medical costs are expected to have a minimal impact on QODD. Given that the original QODD was developed in the United States, some items may not align with medical, cultural, or linguistic practices in Asian countries [12]. In a similar adaptation, six questions were integrated or removed in the Chinese version of the QODD, reducing the questionnaire to 22 items [18]. In the present study, the items (15a) "Did your loved one have one or more visits from a religious or spiritual advisor?” and (16a) "Did your loved one have a spiritual service or ceremony before his/her death?” were revised to use less religious language, adapting it to Japanese culture, which does not strongly express any particular religion. For example, we added the prefatory phrase “when necessary,” because the Japanese are less likely to require the intervention of a religious or spiritual advisor [19].

A strong positive correlation was found between the total and domain scores of the Japanese ICU-QODD and the QODD-1 (range: 0.616-0.930, p<0.001). Previous studies have also reported a correlation between the ICU-QODD score and QODD-1, which supports the convergent validity found in this study [12,20]. Additionally, a known-groups analysis demonstrated that patients admitted to the ICU from a general ward had significantly lower QODD scores (p=0.03), which is consistent with previous findings [21]. No significant differences were observed based on patient gender, chest compressions, or advance directives, which may be attributed to the small sample size. Future studies with larger sample sizes are recommended to further explore these factors.

The Japanese version of ICU-QODD demonstrated strong reliability. Cronbach’s alpha for all domains exceeded 0.7 (range: 0.752-0.995), indicating good internal consistency [22]. The ICC was below 0.7 for the following two items only: the use of dialysis and funeral preparations. The universal health insurance system began covering chronic hemodialysis treatment in 1967. Currently, hemodialysis treatment is almost free of charge, except for those with high incomes (up to 20,000 Japanese Yen per month, or approximately 200 US Dollars) [23]. Consequently, the number of dialysis patients per population in Japan is significantly higher than that of global standards [24]. Dialysis is not only limited to ICU patients but is also relatively common among the public in outpatient and home healthcare settings. Funeral arrangements, as part of advance care planning (ACP), are intended to respect the patient’s wishes and promote emotional well-being [25]. However, the prevalence of ACP in Japan is relatively low, suggesting that making funeral arrangements before death may not be a common practice [26]. Although this Japanese context may have influenced the results, the overall ICC exceeded 0.7 for the total score and other items, supporting its reliability for using Japanese clinical settings.

Strengths and limitations

This study presents the first rigorously developed Japanese version of the ICU-QODD, providing an essential tool for evaluating family perspectives in a culturally appropriate context. A key strength is the standardized translation process, ensuring both validity and reliability. However, several limitations should be noted. First, data were collected from a single hospital ICU in central Japan. Although ICU environments are consistent nationwide owing to Japan’s healthcare system, regional cultural differences could influence responses. Second, while the target sample size was met, the sample size remains relatively small, and caution is needed when interpreting the known-groups analysis results. Third, the low ICCs for dialysis- and funeral-related items warrant further analysis focused on these patients to account for specific patient backgrounds. Finally, this study did not conduct factor analysis for construct validity or evaluate criterion-related validity with other measures. This limitation is attributed to the absence of assessment tools to serve as criteria and the challenge of obtaining an adequate sample size at a single facility for construct factor analysis. However, convergent validity can be confirmed using the QODD-1 as the comparator [20]. Therefore, in this study, validity was assessed by confirming convergent validity rather than by construct validity through factor analysis. Future research should address these aspects to provide a more comprehensive validation.

## Conclusions

A Japanese version of the ICU-QODD was translated, incorporating modifications to align with Japan’s medical, cultural, and linguistic practices. This version was rigorously evaluated for convergent and known-groups validity, internal consistency, and intrarater reliability. The findings indicate that the Japanese ICU-QODD demonstrates adequate validity and reliability, supporting its suitability for using Japanese clinical settings.

## Appendices

Appendix 1

**Table 7:** Original version of the Quality of Dying and Death Questionnaire (QODD). QODD score: the total score for question b is divided by the number of answered items in b, then divided by 10 and multiplied by 100 to get the total score. The total score ranges from 0 to 100, with higher scores indicating a better quality of death and the dying process.

From your perspective, we would like to know how often your loved one had the experiences described below. Please pick a number from 0 to 5 with “0” indicating “none of the time” and “5” indicating “all of the time”. Then, we would like you to rate this aspect of your loved one’s dying experience on a scale from 0 to 10, where “0” is a “terrible experience”, and “10” is an “almost perfect experience”. Please make your best effort to choose a number, even if you are not completely certain of the answer. If you cannot pick a number, please circle “Don’t Know” so that we will know that this is a question you cannot answer. We want you to choose a number based on your experience, not what you think your loved one might have answered.	
1a. How often did your loved one appear to have his/her pain under control? (Circle one number) 0: None of the time, 1: A little bit of the time, 2: Some of the time, 3: A good bit of the time, 4: Most of the time, 5: All of the time, 6: Don’t know >>>>>>>>>> Go to Question 2a.	
b. How would you rate this aspect of your loved one’s dying experience? (Circle one number)	Terrible 0 1 2 3 4 5 6 7 8 9 10 Almost perfect
2a. How often did your loved one appear to have control over what was going on around him/her? (Circle one number) 0: None of the time, 1: A little bit of the time, 2: Some of the time, 3: A good bit of the time, 4: Most of the time, 5: All of the time, 6: Don’t know >>>>>>>>>> Go to Question 3a.	
b. How would you rate this aspect of your loved one’s dying experience? (Circle one number)	Terrible 0 1 2 3 4 5 6 7 8 9 10 Almost perfect
3a. How often was your loved one able to feed her/himself? (Circle one number) 0: None of the time, 1: A little bit of the time, 2: Some of the time, 3: A good bit of the time, 4: Most of the time, 5: All of the time, 6: Don’t know >>>>>>>>>> Go to Question 4a.	
b. How would you rate this aspect of your loved one’s dying experience? (Circle one number)	Terrible 0 1 2 3 4 5 6 7 8 9 10 Almost perfect
4a. How often did your loved one appear to breathe comfortably? (Circle one number) 0: None of the time, 1: A little bit of the time, 2: Some of the time, 3: A good bit of the time, 4: Most of the time, 5: All of the time, 6: Don’t know >>>>>>>>>> Go to Question 5a.	
b. How would you rate this aspect of your loved one’s dying experience? (Circle one number)	Terrible 0 1 2 3 4 5 6 7 8 9 10 Almost perfect
5a. How often did your loved one appear to feel at peace with dying? (Circle one number) 0: None of the time, 1: A little bit of the time, 2: Some of the time, 3: A good bit of the time, 4: Most of the time, 5: All of the time, 6: Don’t know >>>>>>>>>> Go to Question 6a.	
b. How would you rate this aspect of your loved one’s dying experience? (Circle one number)	Terrible 0 1 2 3 4 5 6 7 8 9 10 Almost perfect
6a. How often did your loved one appear to be unafraid of dying? (Circle one number) 0: None of the time, 1: A little bit of the time, 2: Some of the time, 3: A good bit of the time, 4: Most of the time, 5: All of the time, 6: Don’t know >>>>>>>>>> Go to Question 7a.	
b. How would you rate this aspect of your loved one’s dying experience? (Circle one number)	Terrible 0 1 2 3 4 5 6 7 8 9 10 Almost perfect
7a. How often did your loved one laugh and smile? (Circle one number) 0: None of the time, 1: A little bit of the time, 2: Some of the time, 3: A good bit of the time, 4: Most of the time, 5: All of the time, 6: Don’t know >>>>>>>>>> Go to Question 8a.	
b. How would you rate this aspect of your loved one’s dying experience? (Circle one number)	Terrible 0 1 2 3 4 5 6 7 8 9 10 Almost perfect
8a. How often did your loved one appear to keep his/her dignity and self-respect? (Circle one number) 0: None of the time, 1: A little bit of the time, 2: Some of the time, 3: A good bit of the time, 4: Most of the time, 5: All of the time, 6: Don’t know >>>>>>>>>> Go to Question 9a.	
b. How would you rate this aspect of your loved one’s dying experience? (Circle one number)	Terrible 0 1 2 3 4 5 6 7 8 9 10 Almost perfect
9a. How often did your loved one spend time with his/her family or friends? (Circle one number) 0: None of the time, 1: A little bit of the time, 2: Some of the time, 3: A good bit of the time, 4: Most of the time, 5: All of the time, 6: Don’t know >>>>>>>>>> Go to Question 10a.	
b. How would you rate this aspect of your loved one’s dying experience? (Circle one number)	Terrible 0 1 2 3 4 5 6 7 8 9 10 Almost perfect
10a. How often did your loved one spend time alone? (Circle one number) 0: None of the time, 1: A little bit of the time, 2: Some of the time, 3: A good bit of the time, 4: Most of the time, 5: All of the time, 6: Don’t know >>>>>>>>>> Go to Question 11a.	
b. How would you rate this aspect of your loved one’s dying experience? (Circle one number)	Terrible 0 1 2 3 4 5 6 7 8 9 10 Almost perfect
From your perspective, we would like to know how often your loved one had the experiences described below. Please pick a number from 0 to 5 with “0” indicating “none of the time” and “5” indicating “all of the time”. Then, we would like you to rate this aspect of your loved one’s dying experience on a scale from 0 to 10, where “0” is a “terrible experience”, and “10” is an “almost perfect experience”. Please make your best effort to choose a number, even if you are not completely certain of the answer. If you cannot pick a number, please circle “Don’t Know” so that we will know that this is a question you cannot answer. We want you to choose a number based on your experience, not what you think your loved one might have answered.	
1a. How often did your loved one appear to have his/her pain under control? (Circle one number) 0: None of the time, 1: A little bit of the time, 2: Some of the time, 3: A good bit of the time, 4: Most of the time, 5: All of the time, 6: Don’t know >>>>>>>>>> Go to Question 2a.	
b. How would you rate this aspect of your loved one’s dying experience? (Circle one number)	Terrible 0 1 2 3 4 5 6 7 8 9 10 Almost perfect
2a. How often did your loved one appear to have control over what was going on around him/her? (Circle one number) 0: None of the time, 1: A little bit of the time, 2: Some of the time, 3: A good bit of the time, 4: Most of the time, 5: All of the time, 6: Don’t know >>>>>>>>>> Go to Question 3a.	
b. How would you rate this aspect of your loved one’s dying experience? (Circle one number)	Terrible 0 1 2 3 4 5 6 7 8 9 10 Almost perfect
3a. How often was your loved one able to feed her/himself? (Circle one number) 0: None of the time, 1: A little bit of the time, 2: Some of the time, 3: A good bit of the time, 4: Most of the time, 5: All of the time, 6: Don’t know >>>>>>>>>> Go to Question 4a.	
b. How would you rate this aspect of your loved one’s dying experience? (Circle one number)	Terrible 0 1 2 3 4 5 6 7 8 9 10 Almost perfect
4a. How often did your loved one appear to breathe comfortably? (Circle one number) 0: None of the time, 1: A little bit of the time, 2: Some of the time, 3: A good bit of the time, 4: Most of the time, 5: All of the time, 6: Don’t know >>>>>>>>>> Go to Question 5a.	
b. How would you rate this aspect of your loved one’s dying experience? (Circle one number)	Terrible 0 1 2 3 4 5 6 7 8 9 10 Almost perfect
5a. How often did your loved one appear to feel at peace with dying? (Circle one number) 0: None of the time, 1: A little bit of the time, 2: Some of the time, 3: A good bit of the time, 4: Most of the time, 5: All of the time, 6: Don’t know >>>>>>>>>> Go to Question 6a.	
b. How would you rate this aspect of your loved one’s dying experience? (Circle one number)	Terrible 0 1 2 3 4 5 6 7 8 9 10 Almost perfect
6a. How often did your loved one appear to be unafraid of dying? (Circle one number) 0: None of the time, 1: A little bit of the time, 2: Some of the time, 3: A good bit of the time, 4: Most of the time, 5: All of the time, 6: Don’t know >>>>>>>>>> Go to Question 7a.	
b. How would you rate this aspect of your loved one’s dying experience? (Circle one number)	Terrible 0 1 2 3 4 5 6 7 8 9 10 Almost perfect
7a. How often did your loved one laugh and smile? (Circle one number) 0: None of the time, 1: A little bit of the time, 2: Some of the time, 3: A good bit of the time, 4: Most of the time, 5: All of the time, 6: Don’t know >>>>>>>>>> Go to Question 8a.	
b. How would you rate this aspect of your loved one’s dying experience? (Circle one number)	Terrible 0 1 2 3 4 5 6 7 8 9 10 Almost perfect
8a. How often did your loved one appear to keep his/her dignity and self-respect? (Circle one number) 0: None of the time, 1: A little bit of the time, 2: Some of the time, 3: A good bit of the time, 4: Most of the time, 5: All of the time, 6: Don’t know >>>>>>>>>> Go to Question 9a.	
b. How would you rate this aspect of your loved one’s dying experience? (Circle one number)	Terrible 0 1 2 3 4 5 6 7 8 9 10 Almost perfect
9a. How often did your loved one spend time with his/her family or friends? (Circle one number) 0: None of the time, 1: A little bit of the time, 2: Some of the time, 3: A good bit of the time, 4: Most of the time, 5: All of the time, 6: Don’t know >>>>>>>>>> Go to Question 10a.	
b. How would you rate this aspect of your loved one’s dying experience? (Circle one number)	Terrible 0 1 2 3 4 5 6 7 8 9 10 Almost perfect
10a. How often did your loved one spend time alone? (Circle one number) 0: None of the time, 1: A little bit of the time, 2: Some of the time, 3: A good bit of the time, 4: Most of the time, 5: All of the time, 6: Don’t know >>>>>>>>>> Go to Question 11a.	
b. How would you rate this aspect of your loved one’s dying experience? (Circle one number)	Terrible 0 1 2 3 4 5 6 7 8 9 10 Almost perfect
The following questions are answered with either a “Yes” or “No” based on whether your loved one did certain activities. Please rate the quality of that aspect of the dying experience. Again, we are asking you to focus on your loved one’s last several days.	
11a. Was your loved one touched or hugged by his/her loved ones? (Circle one number) 1: Yes, 2: No, 3: Don’t know >>>>>>>>>> Go to Question 12a.	
b. How would you rate this aspect of your loved one’s dying experience? (Circle one number)	Terrible 0 1 2 3 4 5 6 7 8 9 10 Almost perfect
12a. Were all of your loved one’s healthcare costs taken care of? (Circle one number) 1: Yes, 2: No, 3: Don’t know >>>>>>>>>> Go to Question 13a.	
b. How would you rate this aspect of your loved one’s dying experience? (Circle one number)	Terrible 0 1 2 3 4 5 6 7 8 9 10 Almost perfect
13a. Did your loved one say goodbye to loved ones? (Circle one number) 1: Yes, 2: No, 3: Don’t know >>>>>>>>>> Go to Question 14a.	
b. How would you rate this aspect of your loved one’s dying experience? (Circle one number)	Terrible 0 1 2 3 4 5 6 7 8 9 10 Almost perfect
14a. Did your loved one clear up any bad feelings with others? (Circle one number) 1: Yes, 2: No, 3: Don’t know >>>>>>>>>> Go to Question 15a.	
b. How would you rate this aspect of your loved one’s dying experience? (Circle one number)	Terrible 0 1 2 3 4 5 6 7 8 9 10 Almost perfect
15a. Did your loved one have one or more visits from a religious or spiritual advisor? (Circle one number) 1: Yes, 2: No, 3: Don’t know >>>>>>>>>> Go to Question 16a.	
b. How would you rate this aspect of your loved one’s dying experience? (Circle one number)	Terrible 0 1 2 3 4 5 6 7 8 9 10 Almost perfect
16a. Did your loved one have a spiritual service or ceremony before his/her death? (Circle one number) 1: Yes, 2: No, 3: Don’t know >>>>>>>>>> Go to Question 17a.	
b. How would you rate this aspect of your loved one’s dying experience? (Circle one number)	Terrible 0 1 2 3 4 5 6 7 8 9 10 Almost perfect
17a. Did your loved one receive a mechanical ventilator (respirator) to breathe for him/her? (Circle one number) 1: Yes, 2: No, 3: Don’t know >>>>>>>>>> Go to Question 18a.	
b. How would you rate this aspect of your loved one’s dying experience? (Circle one number)	Terrible 0 1 2 3 4 5 6 7 8 9 10 Almost perfect
18a. Did your loved one receive dialysis for his/her kidneys? (Circle one number) 1: Yes, 2: No, 3: Don’t know >>>>>>>>>> Go to Question 19a.	
b. How would you rate this aspect of your loved one’s dying experience? (Circle one number)	Terrible 0 1 2 3 4 5 6 7 8 9 10 Almost perfect
Please answer either “Yes” or “No” if your loved one ever experienced the following. Then, rate the quality of this aspect of your loved one’s dying experience.	
19a. Did your loved one have his or her funeral arrangements in order prior to death? (Circle one number) 1: Yes, 2: No, 3: Don’t know >>>>>>>>>> Go to Question 20a.	
b. How would you rate this aspect of your loved one’s dying experience? (Circle one number)	Terrible 0 1 2 3 4 5 6 7 8 9 10 Almost perfect
20a. Did your loved one discuss his or her wishes for end-of-life care with his/her doctor, for example, resuscitation or intensive care? (Circle one number) 1: Yes, 2: No, 3: Don’t know >>>>>>>>>> Go to Question 21a.	
b. How would you rate this aspect of your loved one’s dying experience? (Circle one number)	Terrible 0 1 2 3 4 5 6 7 8 9 10 Almost perfect
21a. Was anyone present at the moment of your loved one’s death? (Circle one number) 1: Yes, 2: No, 3: Don’t know >>>>>>>>>> Go to Question 22a.	
b. How would you rate this aspect of your loved one’s dying experience? (Circle one number)	Terrible 0 1 2 3 4 5 6 7 8 9 10 Almost perfect
22a. In the moment before your loved one's death what was your loved one's condition? (Circle one number) 1: Awake, 2: Asleep, 3: Unconscious, 4: Don't know >>>>>>>>>> Go to Question 23.	
b. How would you rate this aspect of your loved one’s dying experience? (Circle one number)	Terrible 0 1 2 3 4 5 6 7 8 9 10 Almost perfect
23. Overall, how would you rate the quality of your loved one’s dying? (Circle one number)	Terrible 0 1 2 3 4 5 6 7 8 9 10 Almost perfect
24. Rate the care your loved one received from all doctors and other health care providers (including nurses, caseworkers, and other health care professionals) during the last several days of his or her life while in the ICU. (Circle the number)	Worst healthcare possible 0 1 2 3 4 5 6 7 8 9 10 best healthcare possible
25. Rate the care your loved one received from his or her doctor during the last several days of his or her life while in the ICU. (Circle the number)	Worst healthcare possible 0 1 2 3 4 5 6 7 8 9 10 best healthcare possible

Appendix 2

**Table 8:** Japanese version of the Quality of Dying and Death Questionnaire. QODDの得点： 質問項目bの合計点を回答されたbの項目数で割る。さらに、10で割って100を掛けた数が合計得点となる。合計得点は0～100点で、得点が高いほど死と死にゆく過程の質が高いことを示す。

質問 a. では、あなたの大切な人が、質問内容についてどのくらいの頻度で経験したかを、「0: 全くない」～「5: いつも」でお答えください。あなたの見たことに基づいて数字を選んでください。答えられない場合は、「6：分からない」に〇をつけてください。そして、質問 b. では、その経験（質問aの経験）を「あなたの大切な人はどのように評価したと思うか」について、「0: ひどい」～「10: ほとんど理想的」でお答えください。ここでは、あなたの大切な人がどのように評価したと思うかを想像して、数字を選んでください。	
1a.（あなたから見て）あなたの大切な人は、どのくらい痛みをコントロールできているように見えましたか？（番号を１つ選び 〇 をしてください） 0: 全くない、1: 少し、2: ときどき、3: だいたい、4: ほとんど、5: いつも、6: 分からない>>>>>>>「6: 分からない」を選択した方は質問2 aへ	
b. あなたの大切な人がお亡くなりになる過程で経験したこの点（質問1 a）を、あなたの大切な人はどのように評価したと思いますか？（番号を１つ選び 〇 をしてください）	(ひどい経験) 0 1 2 3 4 5 6 7 8 9 10 (ほとんど理想的な経験)
2a.（あなたから見て）あなたの大切な人は、どのくらい身の回りのことを自分の思う通りにできていましたか？（番号を１つ選び 〇 をしてください） 0: 全くない、1: 少し、2: ときどき、3: だいたい、4: ほとんど、5: いつも、6: 分からない>>>>>>>「6: 分からない」を選択した方は質問3 aへ	
b. あなたの大切な人がお亡くなりになる過程で経験したこの点（質問2 a）を、あなたの大切な人はどのように評価したと思いますか？（番号を１つ選び 〇 をしてください）	(ひどい経験) 0 1 2 3 4 5 6 7 8 9 10 (ほとんど理想的な経験)
3a.（あなたから見て）あなたの大切な人は、どのくらい自分で食事をすることができていましたか？（番号を１つ選び 〇 をしてください） 0: 全くない、1: 少し、2: ときどき、3: だいたい、4: ほとんど、5: いつも、6: 分からない>>>>>>>「6: 分からない」を選択した方は質問4 aへ	
b. あなたの大切な人がお亡くなりになる過程で経験したこの点（質問3 a）を、あなたの大切な人はどのように評価したと思いますか？（番号を１つ選び 〇 をしてください）	(ひどい経験) 0 1 2 3 4 5 6 7 8 9 10 (ほとんど理想的な経験)
4a.（あなたから見て）あなたの大切な人は、どのくらい穏やかに呼吸をしているように見えましたか？（番号を１つ選び 〇 をしてください） 0: 全くない、1: 少し、2: ときどき、3: だいたい、4: ほとんど、5: いつも、6: 分からない>>>>>>>「6: 分からない」を選択した方は質問5 aへ	
b. あなたの大切な人がお亡くなりになる過程で経験したこの点（質問4 a）を、あなたの大切な人はどのように評価したと思いますか？（番号を１つ選び 〇 をしてください）	(ひどい経験) 0 1 2 3 4 5 6 7 8 9 10 (ほとんど理想的な経験)
5a.（あなたから見て）あなたの大切な人は、お亡くなりになるまでの時期をどのくらい穏やかに過ごしているように見えましたか？（番号を１つ選び 〇 をしてください） 0: 全くない、1: 少し、2: ときどき、3: だいたい、4: ほとんど、5: いつも、6: 分からない>>>>>>>「6: 分からない」を選択した方は質問6 aへ	
b. あなたの大切な人がお亡くなりになる過程で経験したこの点（質問5 a）を、あなたの大切な人はどのように評価したと思いますか？（番号を１つ選び 〇 をしてください）	(ひどい経験) 0 1 2 3 4 5 6 7 8 9 10 (ほとんど理想的な経験)
6a.（あなたから見て）あなたの大切な人は、どのくらいお亡くなりになることを恐れていないように見えましたか？（番号を１つ選び 〇 をしてください） 0: 全くない、1: 少し、2: ときどき、3: だいたい、4: ほとんど、5: いつも、6: 分からない>>>>>>>「6: 分からない」を選択した方は質問7 aへ	
b. あなたの大切な人がお亡くなりになる過程で経験したこの点（質問6 a）を、あなたの大切な人はどのように評価したと思いますか？（番号を１つ選び 〇 をしてください）	(ひどい経験) 0 1 2 3 4 5 6 7 8 9 10 (ほとんど理想的な経験)
7a.（あなたから見て）あなたの大切な人は、どのくらい笑ったり、微笑んだりしていましたか？（番号を１つ選び 〇 をしてください） 0: 全くない、1: 少し、2: ときどき、3: だいたい、4: ほとんど、5: いつも、6: 分からない>>>>>>>「6: 分からない」を選択した方は質問8 aへ	
b. あなたの大切な人がお亡くなりになる過程で経験したこの点（質問7 a）を、あなたの大切な人はどのように評価したと思いますか？（番号を１つ選び 〇 をしてください）	(ひどい経験) 0 1 2 3 4 5 6 7 8 9 10 (ほとんど理想的な経験)
8a.（あなたから見て）あなたの大切な人は、どのくらい自尊心を保てたり、人として尊重されたりしているように見えましたか？（番号を１つ選び 〇 をしてください） 0: 全くない、1: 少し、2: ときどき、3: だいたい、4: ほとんど、5: いつも、6: 分からない>>>>>>>「6: 分からない」を選択した方は質問9 aへ	
b. あなたの大切な人がお亡くなりになる過程で経験したこの点（質問8 a）を、あなたの大切な人はどのように評価したと思いますか？（番号を１つ選び 〇 をしてください）	(ひどい経験) 0 1 2 3 4 5 6 7 8 9 10 (ほとんど理想的な経験)
9a.（あなたから見て）あなたの大切な人は、どのくらい家族や友人と過ごしていましたか? （番号を１つ選び 〇 をしてください） 0: 全くない、1: 少し、2: ときどき、3: だいたい、4: ほとんど、5: いつも、6: 分からない>>>>>>>「6: 分からない」を選択した方は質問10 aへ	
b. あなたの大切な人がお亡くなりになる過程で経験したこの点（質問9 a）を、あなたの大切な人はどのように評価したと思いますか？（番号を１つ選び 〇 をしてください）	(ひどい経験) 0 1 2 3 4 5 6 7 8 9 10 (ほとんど理想的な経験)
10a.（あなたから見て）あなたの大切な人は、どのくらいプライベートな時間を過ごしていましたか？（番号を１つ選び 〇 をしてください） 0: 全くない、1: 少し、2: ときどき、3: だいたい、4: ほとんど、5: いつも、6: 分からない>>>>>>>「6: 分からない」を選択した方は質問11 aへ	
b. あなたの大切な人がお亡くなりになる過程で経験したこの点（質問10 a）を、あなたの大切な人はどのように評価したと思いますか？（番号を１つ選び 〇 をしてください）	(ひどい経験) 0 1 2 3 4 5 6 7 8 9 10 (ほとんど理想的な経験)

以下の質問 a. では、あなたの大切な人が経験した活動に基づいて、「はい」または「いいえ」でお答えください。質問 b. では、あなたの大切な人がお亡くなりになる過程で経験したことの質について評価してください。ここでも、あなたの大切な人の最期の数日間に焦点を当ててください。	
11a. あなたの大切な人は、その人にとって大切な人に触られたり、抱きしめられたりしましたか？（番号を１つ選び 〇 をしてください） 1: はい、2: いいえ、3: 分からない>>>>>>>「3: 分からない」を選択した方は質問12 aへ	
b. あなたの大切な人がお亡くなりになる過程で経験したこの点（質問11 a）を、あなたの大切な人はどのように評価したと思いますか？（番号を１つ選び 〇 をしてください）	(ひどい経験) 0 1 2 3 4 5 6 7 8 9 10 (ほとんど理想的な経験)
12a. あなたの大切な人は、親しい人に別れを告げることができましたか？（番号を１つ選び 〇 をしてください） 1: はい、2: いいえ、3: 分からない>>>>>>>「3: 分からない」を選択した方は質問13 aへ	
b. あなたの大切な人がお亡くなりになる過程で経験したこの点（質問12 a）を、あなたの大切な人はどのように評価したと思いますか？（番号を１つ選び 〇 をしてください）	(ひどい経験) 0 1 2 3 4 5 6 7 8 9 10 (ほとんど理想的な経験)
13a. あなたの大切な人は、他者への心残りを解消しましたか？（番号を１つ選び 〇 をしてください） 1: はい、2: いいえ、3: 分からない>>>>>>>「3: 分からない」を選択した方は質問14 aへ	
b. あなたの大切な人がお亡くなりになる過程で経験したこの点（質問13 a）を、あなたの大切な人はどのように評価したと思いますか？（番号を１つ選び 〇 をしてください）	(ひどい経験) 0 1 2 3 4 5 6 7 8 9 10 (ほとんど理想的な経験)
14a. あなたの大切な人は、希望した場合に、宗教的またはスピリチュアルな支えとなる人と1回以上面会しましたか？（番号を１つ選び 〇 をしてください） 1: はい、2: いいえ、3: 分からない>>>>>>>「3: 分からない」を選択した方は質問15 aへ	
b. あなたの大切な人がお亡くなりになる過程で経験したこの点（質問14 a）を、あなたの大切な人はどのように評価したと思いますか？（番号を１つ選び 〇 をしてください）	(ひどい経験) 0 1 2 3 4 5 6 7 8 9 10 (ほとんど理想的な経験)
15a. あなたの大切な人は、希望した場合に、お亡くなりになる前にスピリチュアルなサービスまたはセレモニー（お別れの会なども含む）を受けましたか？（番号を１つ選び 〇 をしてください） 1: はい、2: いいえ、3: 分からない>>>>>>>「3: 分からない」を選択した方は質問16 aへ	
b. あなたの大切な人がお亡くなりになる過程で経験したこの点（質問15 a）を、あなたの大切な人はどのように評価したと思いますか？（番号を１つ選び 〇 をしてください）	(ひどい経験) 0 1 2 3 4 5 6 7 8 9 10 (ほとんど理想的な経験)
16a. あなたの大切な人は、人工呼吸器による呼吸の補助を受けていましたか？（番号を１つ選び 〇 をしてください） 1: はい、2: いいえ、3: 分からない>>>>>>>「3: 分からない」を選択した方は質問17 aへ	
b. あなたの大切な人がお亡くなりになる過程で経験したこの点（質問16 a）を、あなたの大切な人はどのように評価したと思いますか？（番号を１つ選び 〇 をしてください）	(ひどい経験) 0 1 2 3 4 5 6 7 8 9 10 (ほとんど理想的な経験)
17a. あなたの大切な人は、透析を受けていましたか？（番号を１つ選び 〇 をしてください） 1: はい、2: いいえ、3: 分からない>>>>>>>「3: 分からない」を選択した方は質問18 aへ	
b. あなたの大切な人がお亡くなりになる過程で経験したこの点（質問17 a）を、あなたの大切な人はどのように評価したと思いますか？（番号を１つ選び 〇 をしてください）	(ひどい経験) 0 1 2 3 4 5 6 7 8 9 10 (ほとんど理想的な経験)

以下の質問 a. では、あなたの大切な人の経験したことに基づいて、「はい」または「いいえ」でお答えください。質問 b. では、あなたの大切な人がお亡くなりになる過程で経験したことの質について評価してください。	
18a. あなたの大切な人は、生前から葬儀の準備をしていましたか？（番号を１つ選び 〇 をしてください） 1: はい、2: いいえ、3: 分からない>>>>>>>「3: 分からない」を選択した方は質問19 aへ	
b. あなたの大切な人がお亡くなりになる過程で経験したこの点（質問18 a）を、あなたの大切な人はどのように評価したと思いますか？（番号を１つ選び 〇 をしてください）	(ひどい経験) 0 1 2 3 4 5 6 7 8 9 10 (ほとんど理想的な経験)
19a. あなたの大切な人は、終末期医療についての希望を医師と話し合いましたか？（例えば、蘇生をするかや集中治療を行うかなど）（番号を１つ選び 〇 をしてください） 1: はい、2: いいえ、3: 分からない>>>>>>>「3: 分からない」を選択した方は質問20 aへ	
b. あなたの大切な人がお亡くなりになる過程で経験したこの点（質問19 a）を、あなたの大切な人はどのように評価したと思いますか？（番号を１つ選び 〇 をしてください）	(ひどい経験) 0 1 2 3 4 5 6 7 8 9 10 (ほとんど理想的な経験)
20a. あなたの大切な人がお亡くなりになる瞬間に立ち会った人はいますか？（番号を１つ選び 〇 をしてください） 1: はい、2: いいえ、3: 分からない>>>>>>>「3: 分からない」を選択した方は質問21 aへ	
b. あなたの大切な人がお亡くなりになる過程で経験したこの点（質問20 a）を、あなたの大切な人はどのように評価したと思いますか？（番号を１つ選び 〇 をしてください）	(ひどい経験) 0 1 2 3 4 5 6 7 8 9 10 (ほとんど理想的な経験)
21a. あなたの大切な人がお亡くなりになる直前、どのような状態でしたか？（番号を１つ選び 〇 をしてください） 1: 覚醒していた、2: 眠っていた、3: 昏睡状態または意識がなかった、4: 分からない>>>>>>>「4: 分からない」を選択した方は質問22へ	
b. あなたの大切な人がお亡くなりになる過程で経験したこの点（質問21 a）を、あなたの大切な人はどのように評価したと思いますか？（番号を１つ選び 〇 をしてください）	(ひどい経験) 0 1 2 3 4 5 6 7 8 9 10 (ほとんど理想的な経験)
22. 総合的にみて、大切な人が亡くなる過程で経験したことの質（終末期の質）を、あなたはどのように評価しますか？（番号を１つ選び 〇 をしてください）	(ひどい経験) 0 1 2 3 4 5 6 7 8 9 10 (ほとんど理想的な経験)
23. あなたの大切な人がICUに入院していた最期の数日間、全ての医師および医療従事者（看護師やその他の医療専門家を含む全ての医療者）から受けたケアについて評価してください。（番号を１つ選び 〇 をしてください）	(考えられる最悪の医療) 0 1 2 3 4 5 6 7 8 9 10 (考えられる最高の医療)
24. あなたの大切な人がICUに入院していた最期の数日間、主治医から受けたケアについて評価してください。（番号を１つ選び 〇 をしてください）	(考えられる最悪の医療) 0 1 2 3 4 5 6 7 8 9 10 (考えられる最高の医療)

## References

[1] Society of Critical Care Medicine (2024). Critical care statistics. Critical care statistics. Mount Prospect, IL: Soc. Crit. Care Med.

[2] Nagaoka K, Ichimura K (2021). Factors related to nurses’ perception of end-of-life care in intensive care units. [Article in Japanese]. Palliat Care Res.

[3] Tatsuno J, Yamase H, Tado A, Fujita N (2014). Current status of end-of-life care in ICUs in Japan and awareness of medical professionals. [Article in Japanese]. J Jpn Acad Crit Care Nurs.

[4] Choi Y, Park M, Kang DH, Lee J, Moon JY, Ahn H (2019). The quality of dying and death for patients in intensive care units: a single center pilot study. Acute Crit Care.

[5] Hill SA, Dawood A, Boland E, Leahy HE, Murtagh FE (2022). Palliative medicine in the intensive care unit: needs, delivery, quality. BMJ Support Palliat Care.

[6] Naya K, Sakuramoto H, Aikawa G (2024). Intensive care unit interventions to improve quality of dying and death: scoping review. BMJ Support Palliat Care.

[7] Kinoshita S (2017). Quality of care in the intensive care unit (ICU): a review of international literature on family assessment. [Article in Japanese]. J Nurs Kanto Gakuin Univ.

[8] Naya K, Sakuramoto H, Aikawa G (2024). Family members’ feedback on the “quality of death” of adult patients who died in intensive care units and the factors affecting the death quality: a systematic review and meta-analysis. Cureus.

[9] Downey L, Curtis JR, Lafferty WE, Herting JR, Engelberg RA (2010). The Quality of Dying and Death Questionnaire (QODD): empirical domains and theoretical perspectives. J Pain Symptom Manage.

[10] End-of-Life Care Research Program (2023). QODD version 3.2: family member/friend after-death self-administered questionnaire. http://depts.washington.edu/eolcare/products/instruments/.

[11] Sousa VD, Rojjanasrirat W (2011). Translation, adaptation and validation of instruments or scales for use in cross-cultural health care research: a clear and user-friendly guideline. J Eval Clin Pract.

[12] Glavan BJ, Engelberg RA, Downey L, Curtis JR (2008). Using the medical record to evaluate the quality of end-of-life care in the intensive care unit. Crit Care Med.

[13] Zou GY (2012). Sample size formulas for estimating intraclass correlation coefficients with precision and assurance. Stat Med.

[14] Paiva BS, Valentino TC, Mingardi M (2022). Translation, validity and internal consistency of the Quality of Dying and Death Questionnaire for Brazilian families of patients that died from cancer: a cross-sectional and methodological study. Sao Paulo Med J.

[15] Browne MW, Cudeck R (1992). Alternative ways of assessing model fit. Sociol Methods Res.

[16] Koo TK, Li MY (2016). A guideline of selecting and reporting intraclass correlation coefficients for reliability research. J Chiropr Med.

[17] Kanda K, Takashima N, Tsuji Y, Yokoyama K, Hirao T (2019). Quality of dying and death desired by residents of Kagawa Prefecture, Japan: a qualitative study. Environ Health Prev Med.

[18] Han XP, Mei X, Zhang J, Zhang TT, Yin AN, Qiu F, Liu MJ (2021). Validation of the Chinese version of the Quality of Dying and Death Questionnaire for family members of ICU patients. J Pain Symptom Manage.

[19] Chakraborty R, El-Jawahri AR, Litzow MR, Syrjala KL, Parnes AD, Hashmi SK (2017). A systematic review of religious beliefs about major end-of-life issues in the five major world religions. Palliat Support Care.

[20] Cronbach LJ (1951). Coefficient alpha and the internal structure of tests. Psychometrika.

[21] Cho JY, Lee J, Lee SM (2018). Transcultural adaptation and validation of Quality of Dying and Death Questionnaire in medical intensive care units in South Korea. Acute Crit Care.

[22] Long AC, Kross EK, Engelberg RA, Downey L, Nielsen EL, Back AL, Curtis JR (2014). Quality of dying in the ICU: is it worse for patients admitted from the hospital ward compared to those admitted from the emergency department?. Intensive Care Med.

[23] Hanafusa N, Fukagawa M (2020). Global dialysis perspective: Japan. Kidney360.

[24] Hanafusa N, Abe Met, Joki N (2024). Annual dialysis data report 2020, JSDT renal data registry. Ren Replace Ther.

[25] Winnifrith T, Millington-Sanders C, Husbands E, Haros J, Ballinger H (2024). Proactive advance care planning conversations in general practice: a quality improvement project. BMJ Open Qual.

[26] Mori M (2024). Advance care planning - what is the desirable ACP in Japan?. Igaku No Ayumi.

